# Is it really sinus tachycardia?

**DOI:** 10.3402/jchimp.v1i2.7241

**Published:** 2011-07-18

**Authors:** Marc Mugmon

**Affiliations:** Department of Medicine, Union Memorial Hospital, Baltimore, MD, USA

A 56-year-old male with a history of hypertension and coronary disease was admitted after complaining of mild shortness of breath and palpitations. He had undergone coronary bypass surgery 4 years prior to admission and had been followed regularly by his physicians. His resting heart rate at his last office visit <6 months before was 58/min and his hypertensive regimen, consisting of lisinopril only, was recently intensified. He had been under severe stress recently and had not been eating well. His caffeine intake was limited to one large coffee daily.

A left pleural effusion marked his postoperative course after bypass 4 years before, and he underwent thoracentesis without reaccumulation of the fluid. He also had what was felt to have been either atrial flutter or AV nodal re-entrant tachycardia (AVNRT), which reverted to sinus rhythm with a dose of adenosine, followed by 3 months of amiodarone. He reported no recurrent palpitations until this admission. His only other medication was aspirin. His initial blood pressure was 150/85, pulse was 125 and regular, and respirations were 18. He was afebrile. His O_2_ saturation was 99%. The physical exam was unremarkable except for tachycardia. No murmurs, gallops, or rubs were present. His labs were: Hct 52.6 WBC 11,000 Na 139 K 3.8 BUN 16 Creatinine 0.65. The chest X-ray showed no cardiomegaly or vascular congestion and possible left basilar atelectasis.

**Fig. 1:**
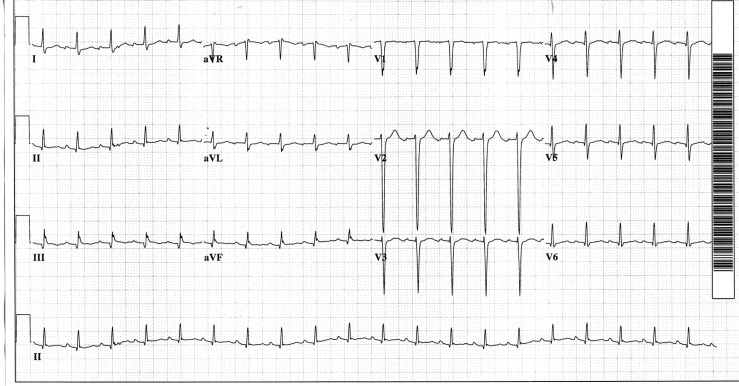
Tracing suggesting sinus tachycardia.

Echocardiography revealed mild concentric left ventricular hypertrophy and some evidence of reduced diastolic compliance, but no pericardial effusion or other abnormalities. The initial electrocardiogram (Fig. 1) was interpreted as sinus tachycardia at a rate of 125. The P-wave morphology was normal and the P-wave axis was normal, both consistent with sinus tachycardia.

Initial diagnostic measures were directed toward the usual causes of sinus tachycardia, such as anemia, dehydration, fever, pulmonary issues (pneumonia, pulmonary embolism), and thyroid disease. The relatively high hematocrit suggested dehydration and he was given intravenous fluids, but no significant heart rate drop was noted. Metoprolol 50 mg orally was administered in the emergency department and he received one 5 mg intravenous dose 8 hours later. Soon thereafter the heart rate dropped to 55 and he became asymptomatic.

**Fig. 2:**
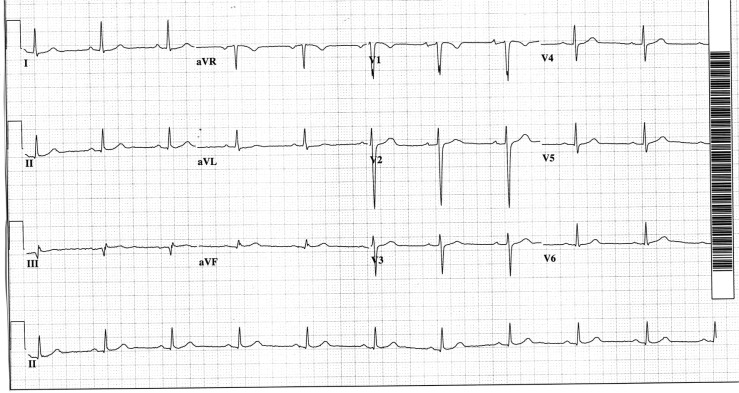
Sinus rhythm replaces what was initially considered to be sinus tachycardia.

A follow-up electrocardiogram (Fig. 2) revealed sinus bradycardia, the only apparent difference from the admission tracing being the rate. The P-wave axis changed slightly but still was in the normal range.

**Fig. 3:**
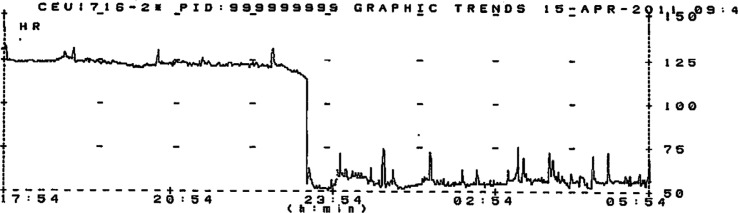
Graphic trend of heart rate demonstrates an abrupt, rather than a gradual drop in heart rate from greater than 100/minute to 60/minute.

The graphic trend from telemetry is seen in Fig. 3. The rate dropped abruptly from 125 to <60, suggesting that the initial rhythm was sinoatrial re-entrant tachycardia (SANRT) rather than sinus tachycardia. The patient was discharged on low dose beta-blocker therapy.

While the monitor did not capture the rhythm at the exact time of the abrupt drop in heart rate, the graphic trend monitor proved crucial in confirming the fact that the rate drop did indeed occur abruptly, and not gradually, as would be expected in sinus rhythm. Based on this observation, a diagnosis of SANRT was felt to be very likely.

The concept of SANRT was postulated in 1943, when Barker suggested the presence of circus rhythms within the sinus node (1). In SANRT, a re-entry circuit is localized to the SA node. This results in a P-wave that has normal morphology, occurring before a regular, narrow QRS complex. The only way it is possible to distinguish it from sinus tachycardia would be an observation of an abrupt onset or termination, which also occurs in other re-entry tachycardias, especially typical AV nodal re-entrant tachycardia (AVNRT). SANRT may respond to vagal maneuvers, and the fact that this patient had a similar episode after his bypass procedure that responded to adenosine suggests that it had not been atrial flutter then but more likely SANRT.

SANRT is usually not associated with severe symptoms. One study demonstrated SANRT in <2% of 379 patients undergoing diagnostic electrophysiologic study (2). However, one author found a nearly 17% incidence in patients being studied, almost all of whom had concomitant organic heart disease (3). Since many patients are asymptomatic, SANRT may be more common than appreciated.

Clinically, the only way to distinguish SANRT from sinus tachycardia is by recognizing its abrupt onset and termination. Sinus tachycardia would manifest a gradual change in rate. Other supraventricular tachyarrhythmias, such as atrial flutter and AVNRT, would demonstrate P-wave morphologies that differ from normal. The rate in SANRT is typically 100–150.

Vagal maneuvers may be effective in terminating SANRT. Carotid massage or Valsalva maneuver may convert SANRT to sinus. Because of the relatively low frequency of the arrhythmia, no large pharmacologic studies are available and therapy is usually empiric and includes beta blockers, amiodarone, verapamil, and/or digitalis. Radiofrequency ablation can also be effective and may allow freedom from antiarrhythmic agents (4).
